# Women’s Experience with Non-Invasive Prenatal Testing and Emotional Well-being and Satisfaction after Test-Results

**DOI:** 10.1007/s10897-017-0118-3

**Published:** 2017-06-30

**Authors:** Rachèl V. van Schendel, G. C. M. Lieve Page-Christiaens, Lean Beulen, Caterina M. Bilardo, Marjon A. de Boer, Audrey B. C. Coumans, Brigitte H. W. Faas, Irene M. van Langen, Klaske D. Lichtenbelt, Merel C. van Maarle, Merryn V. E. Macville, Dick Oepkes, Eva Pajkrt, Lidewij Henneman, for the Dutch NIPT Consortium

**Affiliations:** 1 Department of Clinical Genetics, Section Community Genetics and Amsterdam Public Health Research Institute, VU University Medical Center, PO Box 7057, 1007 MB Amsterdam, The Netherlands; 2 Department Obstetrics and Gynaecology, University Medical Center Utrecht, Utrecht, The Netherlands; 3 Department of Obstetrics and Gynaecology, Radboud University Medical Center, Nijmegen, The Netherlands; 4 Fetal Medicine Unit, Department of Obstetrics and Gynaecology, University Medical Center Groningen, University of Groningen, Groningen, the Netherlands; 5 Department of Obstetrics and Gynaecology, VU University Medical Center, Amsterdam, the Netherlands; 6 Department of Obstetrics and Gynaecology, Maastricht UMC +, Maastricht, the Netherlands; 7 Department of Human Genetics, Radboud University Medical Center, Nijmegen, the Netherlands; 8 Department of Genetics, University Medical Center Groningen, University of Groningen, Groningen, the Netherlands; 9 Department of Medical Genetics, University Medical Center Utrecht, Utrecht, the Netherlands; 10 Department of Clinical Genetics, Academic Medical Center, Amsterdam, the Netherlands; 11 Department of Clinical Genetics, Maastricht UMC +, Maastricht, the Netherlands; 12 Department of Obstetrics, Leiden University Medical Center, Leiden, the Netherlands; 13 Fetal Medicine Unit, Department of Obstetrics and Gynaecology, Academic Medical Centre, Amsterdam, the Netherlands

**Keywords:** Prenatal Screening, NIPT, Non-invasive Prenatal Testing, Anxiety, Reassurance, Satisfaction

## Abstract

Increasingly, high-risk pregnant women opt for non-invasive prenatal testing (NIPT) instead of invasive diagnostic testing. Since NIPT is less accurate than invasive testing, a normal NIPT result might leave women less reassured. A questionnaire study was performed among pregnant women with elevated risk for fetal aneuploidy based on first-trimester combined test (risk ≥1:200) or medical history, who were offered NIPT in the nationwide Dutch TRIDENT study. Pre- and post-test questionnaires (*n* = 682) included measures on: experiences with NIPT procedure, feelings of reassurance, anxiety (State-Trait Anxiety Inventory, STAI), child-related anxiety (PRAQ-R), and satisfaction. The majority (96.1%) were glad to have been offered NIPT. Most (68.5%) perceived the waiting time for NIPT results (mean: 15 days, range 5–32) as (much) too long. Most women with a normal NIPT result felt reassured (80.9%) or somewhat reassured (15.7%). Levels of anxiety and child-related anxiety were significantly lower after receiving a normal NIPT result as compared to the moment of intake (*p* < 0.001). Women with inadequate health literacy or a medical history (e.g. previous child with trisomy) experienced significantly higher post-test-result anxiety (Mean (M) STAI = 31.6 and 30.0, respectively) compared to those with adequate health literacy (M = 28.6) and no medical history (M = 28.6), indicating these women might benefit from extra information and/or guidance when communicating NIPT test-results. Introducing NIPT as an alternative to invasive testing, led to an offer that satisfied and largely reassured high-risk pregnant women.

**Electronic supplementary material:**

The online version of this article (doi:10.1007/s10897-017-0118-3) contains supplementary material, which is available to authorized users.

## Introduction

Since 2011, cell-free DNA (cfDNA) testing for the detection of fetal aneuploidy, also called non-invasive prenatal testing (NIPT), has become widely available for pregnant women, including those at elevated risk for aneuploidy based on the first-trimester combined test (FCT). Until recently, the only choice for these women was between refraining from further testing or having invasive testing (amniocentesis or chorionic villus sampling) to confirm whether their child indeed had an aneuploidy. Because invasive tests have a small procedure-related miscarriage risk (Tabor et al. 2010), many women declined these tests (Nakata et al. 2010), whilst those accepting testing experienced considerable anxiety (Nakic Rados et al. 2013; Sarkar et al. 2006). With the introduction of NIPT, high-risk women are offered a safe and highly sensitive second-tier test (>99% for trisomy 21) (Gil et al. 2015). Due to the small possibility of false positives, abnormal NIPT results still need to be confirmed with invasive testing (Dondorp et al. 2015; ACMG 2013).

Elevated levels of stress and anxiety during pregnancy are associated with potential adverse obstetrical outcomes such as preterm delivery and reduced birth weight (Mancuso et al. 2004; Mulder et al. 2002), and are therefore important to avoid. The mere fact of having a prenatal test for fetal abnormalities may affect maternal anxiety (Nakic Rados et al. 2013). Experienced levels of anxiety further increase upon receiving a high-risk result (Lou et al. 2015). Anxiety is inversely correlated with, amongst others, socio-economic status (Faisal-Cury et al. 2007), but might be contained by promoting informed choice for testing (Michie et al. 2002).

The safety and non-invasiveness of NIPT, which are seen as a great advantage by pregnant women (Lewis et al. 2013), will likely cause less anxiety than a risky invasive test would. Since NIPT is less accurate than invasive testing, however, a normal NIPT result might leave women feeling less reassured. Moreover, NIPT performed using whole-genome sequencing can lead to fetal and/or maternal secondary findings (Brady et al. 2016), which might cause uncertainty. Studies amongst high-risk women undergoing confirmative invasive testing showed that their high levels of anxiety faded away after obtaining a normal result (Lou et al. 2015). With regard to NIPT the literature shows mixed results, with some studies (Vanstone et al. 2015; Wittman et al. 2016) finding a negative (favorable) NIPT result is perceived as being reassuring, but others, although hypothetically addressed (Allyse et al. 2014), have shown women still want further reassurance through invasive testing. As part of an implementation study on NIPT in the UK National Health Service, Lewis et al. (2016) showed a reduction in elevated anxiety levels from 30% to 14% after NIPT results (most of them being negative) had been received.

Although several studies on the uptake of NIPT among high-risk pregnant women have appeared (Chetty et al. 2013; Taylor et al. 2014), more needs to be known about how women experience the offer of NIPT within a national prenatal care setting, and if they are satisfied with the procedure. In the UK women at elevated risk who underwent NIPT in the maternity care pathway of the National Health Services experienced little regret and were overwhelmingly positive about NIPT (Lewis et al. 2016). Here we studied Dutch high-risk women who were offered NIPT as an alternative to invasive testing within the nationwide TRIDENT study (Trial by Dutch laboratories for Evaluation of Non-Invasive Prenatal Testing) (Oepkes et al. 2016). Previously we have shown that 78% of women had made an informed choice for NIPT (van Schendel et al. 2016). Informed choice is defined as a decision made with sufficient knowledge, consistent with the decision-maker’s values and behaviorally implemented (Michie et al. 2002). Informed choice was associated with less decisional conflict and less pre-test anxiety (van Schendel et al. 2016).

In this part of the study, the following questions were addressed: 1) How do women experience the NIPT offer and procedure (e.g. waiting time)? 2) Do women feel reassured and less anxious after receiving a favorable NIPT result and which characteristics of women are associated with higher levels of anxiety? 3) Do women feel satisfied with their choice for NIPT?, and 4) Who do they think should be offered NIPT in the future?

## Methods

### Procedure and Participants

The TRIDENT study evaluated the introduction of NIPT in a governmentally supported and public healthcare-funded prenatal Down syndrome screening program (Oepkes et al. 2016). All women with an elevated risk for fetal aneuploidy based on FTC (cut-off risk ≥1:200) or based on medical history (e.g. prior pregnancy with a fetal trisomy) were referred for further counseling to one of the eight Centers for Prenatal Diagnosis, or one of their satellite centers. Women were offered a choice between NIPT, invasive testing or no further testing. Inclusion and exclusion criteria are described elsewhere (Oepkes et al. 2016). All women were given oral counseling by obstetricians, maternal fetal medicine specialists or specially trained counselors, and a leaflet. Women were counseled about test procedures; reporting time; test sensitivity and specificity for trisomies 21, 18 and 13; and the necessity to confirm abnormal NIPT results with invasive testing. The miscarriage risk for amniocentesis and chorionic villus sampling was mentioned to be 0.3% and 0.5% respectively. All counselors had followed a nationwide uniform training program and were certified after passing a test. Patient information materials were uniform for all centers. All study information was also available online (www.meerovernipt.nl).

Whole-genome massively parallel sequencing was used for NIPT. Results were communicated as “normal” result for aneuploidy (<1:1000 residual risk of aneuploidy, invasive testing not indicated) or “abnormal result” (high-risk for trisomy 21, 18 or 13, or for other chromosomal abnormalities), requiring confirmation with invasive testing. Results were given either by telephone, SMS or mail.

During the first five months of the TRIDENT study (April 1st - September 1st 2014) all pregnant women reporting for counseling because of an elevated FCT risk or medical history in 7 out of the 8 centers (or their satellite centers) were asked by their counselor to fill out two questionnaires. The first questionnaire (Q1) assessed women’s preferences and decision-making and was filled out at intake after NIPT counseling. Results have been presented elsewhere (van Schendel et al. 2016). The second questionnaire (Q2) was filled out after NIPT or invasive test-results were received. Outcome of women who chose to have NIPT will be presented here. Approval for the study was granted by the Dutch government through a Population Screening Act License (No. 350010–118,701-PG) and by local University Medical Ethics Committees.

### Measures

A combination of validated measures in the questionnaire were used to measure anxiety as well as questions context-specific to the TRIDENT study to assess experiences, reassurance and satisfaction that were developed specifically for this questionnaire for the purpose of informing clinical practice rather than for explaining a phenomenon. The questionnaires were designed by a multidisciplinary team of social scientists, psychologists, obstetricians, and a clinical geneticist.

#### Experience with Test Offer and Procedure

Women were asked to respond to a statement on the offer of NIPT: ‘I am happy that NIPT was offered to me’, and on the time they needed to make a decision about NIPT: ‘I had sufficient time to make my choice about (follow-up) testing’ (completely disagree (1)-completely agree (5)). Both scales were compressed to a 3-point scale. Women were also asked if they had a difficult time agreeing about NIPT with their partner (yes; no; not applicable).

Women were asked how many days they had to wait for their test-results and how they felt about the length of the waiting time (way too long (1)-way too short (5)). It was assessed in what way they had received their test-result, and how they would have preferred to receive it (answer options: by mail; by telephone; by email; by SMS; by Patient Portal; by doctor’s consultation; other).

#### NIPT Test-Result

Women were asked what their NIPT test-result was (normal result; abnormal (high-risk) result trisomy 21; trisomy 18; or trisomy 13; or high-risk result for another chromosomal abnormality).

#### Anxiety

Anxiety was measured by a Dutch version of the six-item short form of the state scale of the Spielberger State-Trait Anxiety Inventory (STAI) (Marteau & Bekker 1992; van der Bij et al. 2003). Women were scored on a scale of 20–80, where higher scores mean higher levels of anxiety. A STAI score of 34–36 is considered normal anxiety (Bekker et al. 2003), with pregnant women that choose to have a test having slightly higher STAI scores (Green et al. 2004). Scale reliability was good with a Cronbach’s alpha of 0.87.

Child-related anxiety was measured by the subscale ‘fear of bearing a handicapped child’ (four items) of the Pregnancy-Related Anxiety Questionnaire-Revised (PRAQ-R scale) (Huizink et al. 2004). Women scored on a scale of 4–20, where higher scores mean higher levels of child-related anxiety. Cronbach’s alpha was 0.90.

STAI and PRAQ-R were measured in both questionnaires to compare levels of anxiety before testing and after receiving test-results (Q1 and Q2).

#### Reassurance

Three single-items evaluated whether women felt reassured by a normal NIPT result: *‘*I felt reassured by the test-result’, ‘I am confident that the test-result is correct’ and ‘The test-result offers me sufficient certainty whether my child has a disorder’ (not at all applicable (1)-very much applicable (4)). Women were also asked if they had had confirmative invasive testing after their normal NIPT result or were considering doing.

#### Satisfaction

Women were asked if, retrospectively, they regretted having had NIPT (not at all applicable (1)-very much applicable (4)) and if they would have preferred to have a test different from NIPT (yes; no). They were also asked if they would have preferred to receive test-results earlier in pregnancy (not at all applicable (1)-very much applicable (4)).

#### Future Offer of NIPT

Women were asked to whom they thought NIPT should be offered (answer options (more than one answer allowed): all pregnant women irrespective of their risk for aneuploidy; pregnant women who are at high risk based on the FCT; pregnant women who are ≥36 years old; pregnant women who had an earlier pregnancy with aneuploidy; nobody; other).

#### Women’s Characteristics

The following sociodemographic variables were assessed (from Q1): age, level of education, ethnicity and religion. Women were asked to specify their gestational age and parity. Health literacy was measured by a Dutch version of Chew’s Set of Brief Screening Questions (SBSQ) (Chew et al. 2004). Informed decision-making regarding testing with NIPT was assessed from Q1 (van Schendel et al. 2016) and based on the Multidimensional Measure of Informed Choice (MMIC) (Michie et al. 2002).

### Data Analysis

Descriptive analyses were used to describe women’s characteristics and responses to statements measuring experiences with NIPT test offer and procedure, reassurance, satisfaction, and whom to offer NIPT. Due to non-normal distribution of the data, Wilcoxon paired signed rank tests were used to measure the difference in STAI- and PRAQ-R scores at intake versus after receiving test-results. A multiple regression model using analysis of covariance (ANCOVA) (backward elimination method) looked at the variables associated with higher ‘post-test-result STAI scores’ and higher ‘post-test-result PRAQ-R scores’, while correcting for the covariates: ‘pre-test STAI scores’ and ‘pre-test PRAQ-R scores’. All analyses were performed using SPSS version 20 for Windows (IBM Statistics for Windows, NY, USA).

## Results

### Sample Characteristics

In total 708/1253 (response 57%) pregnant women filled out Q2. Non-response was higher among women with low level of education and/or inadequate health literacy (*p* < 0.01), and among women of non-Western ethnicity compared to Dutch women (*p* < 0.01). Thirteen women who had not filled out the first questionnaire (Q1) were excluded because baseline data for STAI and PRAQ-R scores were not available, as were thirteen women who chose to have immediate invasive testing. This resulted in a total sample of 682 women. Women’s characteristics are presented in Table 1. Mean age was 35.8 years (range 22–45) and 64.2% were highly educated. Mean gestational age was 16.9 weeks (range 11–38). For 86.5% the indication for having NIPT was an elevated FCT risk (≥1:200) and for 13.5% a medical history.

**Table 1:** Characteristics of participants, *N* = 682

Characteristics	*n* (%)
Age (years), *missing = 1*	
≤ 25	11 (1.6)
26–35	285 (41.9)
≥ 36	385 (56.5)
Level of education^a^	
Low	42 (6.2)
Intermediate	202 (29.6)
High	438 (64.2)
Ethnicity^b^ *, missing = 5*	
Dutch	528 (78.0)
Other Western	75 (11.1)
Non-Western	74 (10.9)
Religion^c^ *, missing = 2*	
None	441 (64.9)
Christian	201 (29.6)
Muslim	16 (2.4)
Other	22 (3.2)
Health literacy^d^ *, missing = 1*	
Inadequate	46 (6.8)
Adequate	635 (93.2)
Gestational age (weeks), *missing = 2*	
10–20	623 (91.6)
20–30	45 (6.6)
30–40	5 (0.7)
No longer pregnant (miscarriage/TOP)	7 (1.0)
Parity, *missing = 5*	
nulliparous	407 (37.7)
multiparous	672 (62.3)
Indication for NIPT, *missing = 1*	
Elevated risk FCT	589 (86.5)
Medical history^e^	92 (13.5)
A priori risk (FCT risk), *missing = 24*, *NA = 92*	
≥ 1:10	30 (5.3)
1:11–1:100	267 (47.2)
1:101–1:200	267 (47.2)
Test-result NIPT	
Normal result	656 (96.2)
Trisomy 21	14 (2.1)
Trisomy 18	3 (0.4)
Trisomy 13	1 (0.1)
Other trisomy	8 (1.2)

*TOP* termination of pregnancy, *FCT* first trimester combined test, *NA* not applicable

^a^Low: elementary school, lower level of secondary school, lower vocational training; Medium: higher level of secondary school, intermediate vocational training, High: high vocational training, university (Statistics Netherlands 2016)

^b^Ethnicity was categorized as Dutch, Other Western or Non-Western by the following algorithm: Dutch if both parents were born in the Netherlands; Other Western if at least one of their parents was born in Europe (excluding Turkey), North-America, Oceania, Indonesia or Japan; and non-Western if at least one of their parents was born in Africa, Latin-America, Asia (excluding Indonesia and Japan) and Turkey. If both parents were born abroad, then by country of the mother (Statistics Netherlands 2016)

^c^Christian: Calvinism, Protestantism, Roman-Catholic, Reformed, Baptism. Other: e.g. Jewish, Hindu, Buddhist

^d^Inadequate health literacy if answered other than ‘never’ or ‘occasionally’ on one or more items, based on (Chew et al. 2004)

^e^E.g. a previous child with a trisomy 21, 18 or 13

In total, 656 women (96.2%) reported to have received a normal NIPT result, 14 women (2.1%) reported a high-risk (abnormal) NIPT result for trisomy 21, three women (0.4%) for trisomy 18, and one woman (0.1%) for trisomy 13. Furthermore, eight women (1.2%) received a high-risk result for other trisomies. At the time of questionnaire completion (Q2), 14 of these 26 women had had confirmative invasive testing (details see Supplementary Table S1).

### Experiences with Test Offer and Procedure

The vast majority of women (96.1%) stated they were glad to have been offered NIPT. Most women (85.9%) felt they had had sufficient time to reflect on their choice. The vast majority (99.1%) stated that agreeing with their partner about NIPT had been easy.

**Fig. 1:**
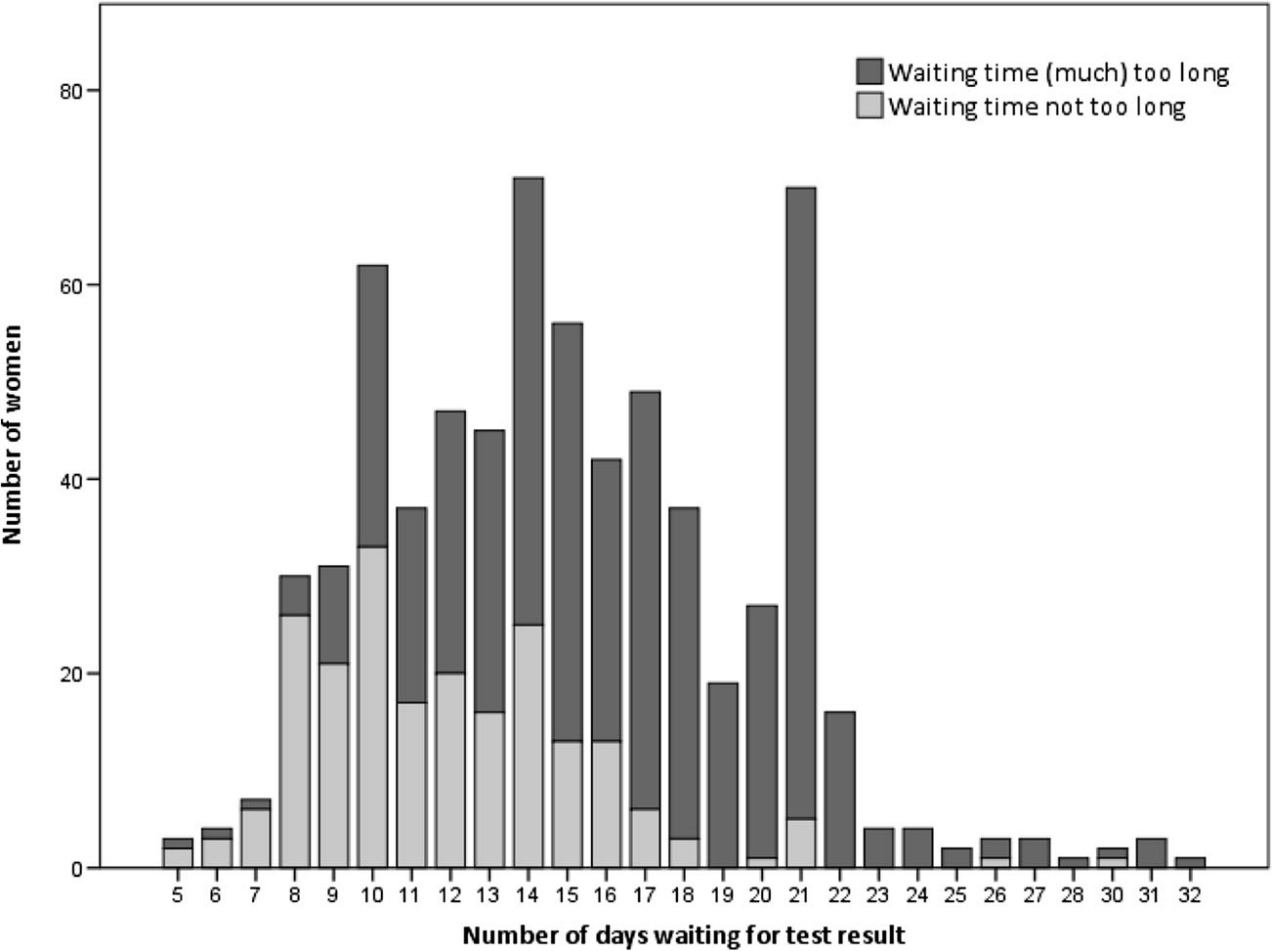
Women’s feelings towards the time waiting for NIPT results, *N* = 682

The mean number of days women reported to have waited for their NIPT result was 15 (range 5–32). 68.5% of women felt that the waiting time was (much) too long and 31.5% thought it was neither too long nor too short. As shown in Fig. 1, a waiting time of ≤10 days was considered acceptable for most women, after this period it was considered too long by the majority of women. Most women indicated that they had received their test-results by telephone (51.8%) or by SMS (23.3%), while 56.1% indicated that telephone was their preferred way for receiving test-results, and only 9.5% stated that SMS was their preferred communication medium (see Supplementary Table S2).

### Anxiety

**Fig. 2:**
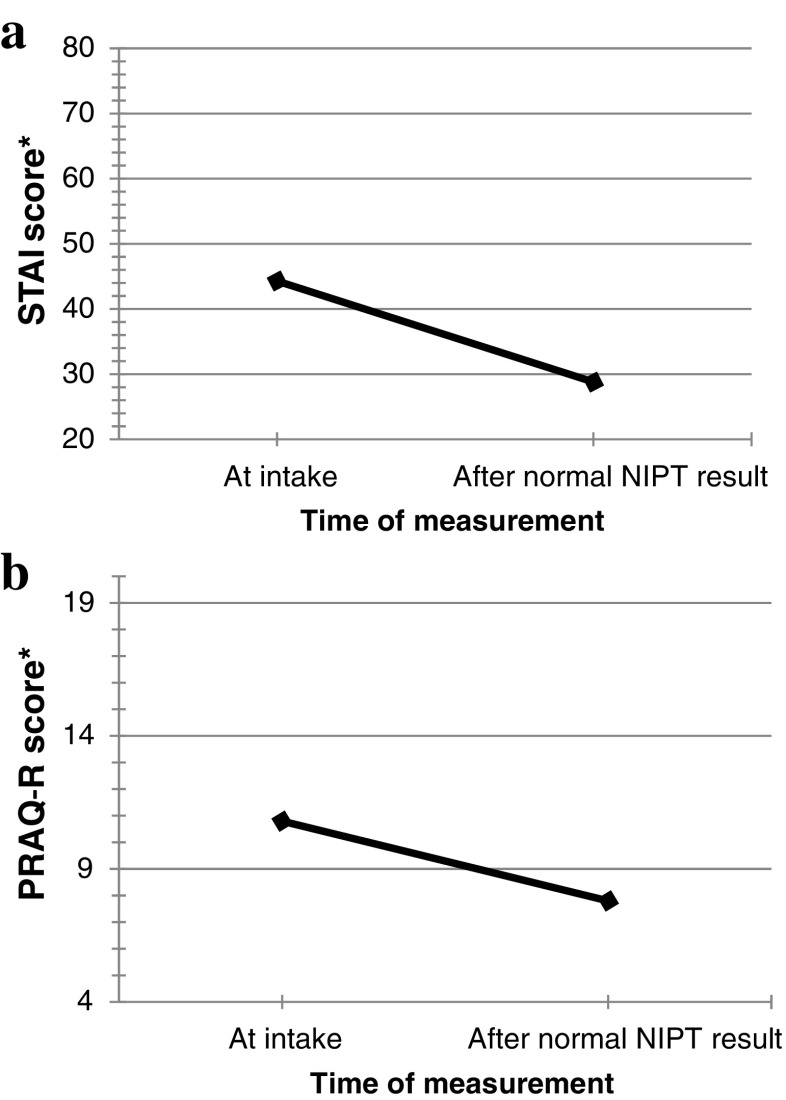
**a** Mean STAI scores at intake (Q1) and after receiving a normal NIPT test-result (Q2), *N* = 656. *Total STAI score (range 20–80). **b** Mean PRAQ-R scores at intake (Q1) and after receiving a normal NIPT test-result (Q2), *N* = 653. *Total PRAQ-R score calculated with subscale ‘child-related anxiety’ (range 4–20)

Women with a normal NIPT result had significantly lower anxiety (STAI) scores after receiving test-results (Mean (M) = 28.8) compared to the anxiety scores at the moment of intake (M = 44.3) (Z = −19.52, *p* < 0.001) (Fig. 2a). For 20 women (3%), anxiety scores were elevated (≥50). ANCOVA analysis revealed that women’s anxiety scores after test-results were significantly related to women’s anxiety scores at intake (*p* < 0.001), meaning that women who had more anxiety beforehand were likely to experience higher anxiety after receiving test-results. The ANCOVA analysis (Table 2) showed that, when controlling for intake STAI score (covariate), women with a normal NIPT result who had inadequate health literacy showed significantly higher STAI scores after test-results (M = 31.6) than women with adequate health literacy (M = 28.6). This was also observed in women who had NIPT based on a medical indication (M = 30.0) compared to those with no medical history (M = 28.6). Age, level of education, a priori risk at FCT and whether or not women had made an informed choice for NIPT had no effect on post-test-result STAI scores. Similar results were shown for child-related anxiety (PRAQ-R) (Table 2). Women with a normal NIPT result showed a significant decrease in level of child-related anxiety, with mean PRAQ-R scores of 10.8 at the moment of intake and mean PRAQ-R scores of 7.8 after receiving test-results (Z = −17.41, *p* < 0.001) (Fig. 2b).

**Table 2:** Multiple regression model using ANCOVA test to identify variables associated with higher post-test-result STAI and PRAQ-R scores in women with a normal NIPT result

Independent Variables	Post-test-result STAI-score (*n* = 320)^a^				Post-test-result PRAQ-R-score (*n* = 320)^a^			
	*d.f*	*Mean square*	*F-ratio*	*P-value* ^*b*^	*d.f*	*Mean square*	*F-ratio*	*P-value* ^*b*^
Age	2	57.229	1.029	0.358	2	3.636	0.539	0.584
Level of education	2	5.949	0.120	0.887	2	15.655	2.330	0.099
A priori risk	2	7.448	0.152	0.859	2	7.569	1.247	0.289
Informed choice	1	69.228	1.244	0.265	1	4.047	0.602	0.438
Health literacy	1	220.478	3.961	**0.047**	1	105.554	15.606	**0.000**
Medical indication for NIPT	1	359.781	6.463	**0.011**	1	47.570	7.033	**0.008**
***Covariate:***								
Pre-test STAI score/Pre-test PRAQ-R score	1	3242.935	58.257	**<0.001**	1	625.675	92.508	**<0.001**

*STAI* State-Trait Anxiety Inventory (Marteau & Bekker 1992; van der Bij et al. 2003), *PRAQ-R* Pregnancy-Related Anxiety Questionnaire-Revised (Huizink et al. 2004)

^a^ *N* = 361 women were excluded from analyses (*n* = 263 to fit criteria of informed choice analysis (van Schendel et al. 2016) and *n* = 98 because of missing values on one of the variables)

^b^Statistical significance set at *p* < 0.05

Overall, the 26 women who had received a high-risk NIPT result for trisomies 21, 18 or 13 or for other trisomies showed high anxiety (STAI) scores after receiving test-results (M = 54.0). For 11 of 14 women who had had confirmatory invasive testing anxiety levels remained high (M ≥ 50.0) (diagnostic testing confirmed that the fetus had a trisomy in 10/11 women). Due to the low number of women receiving each type of result, subgroup analyses are not considered meaningful.

### Reassurance after a Normal NIPT Result

Most women who received a normal NIPT result (80.9%) indicated that the statement ‘I was reassured by the test-result’ very much applied to them, for 15.7% this was somewhat applicable, and for 3.3% it was not (Table 3). Also most women (80.9%) felt confidence that the test-result was correct, while for 18.3% this was somewhat applicable, and for 0.8% it was not. The statement ‘the test result offers me sufficient certainty whether my child has a disorder’ was very much applicable for 64.3% of women, somewhat applicable for 34% of women, and for 1.7% it was not.

**Table 3:** Responses of women with a normal NIPT result (*N* = 656) to statements about reassurance after NIPT results

	Not at all applicable *n* (%)	Hardly applicable *n* (%)	Somewhat applicable *n* (%)	Very much applicable *n* (%)
I was reassured by the test-result	16 (2.4)	6 (0.9)	103 (15.7)	530 (80.9)
I am confident that the test-result is correct	1 (0.2)	4 (0.6)	120 (18.3)	531 (80.9)
The test-result offers me sufficient certainty whether my child has a disorder	2 (0.3)	9 (1.4)	223 (34.0)	421 (64.3)

Numbers may not add up to the total due to missing values

Only two out of 656 women with a normal NIPT result reported to have had confirmatory invasive testing; one because the NIPT result was considered less reliable due to her high body weight and the other woman because a fetal anomaly was suspected at later ultrasound examination.

### Satisfaction

The vast majority of women (97.5%) did not regret having NIPT. About a third of women (28.6%) very much agreed that they would have preferred to receive NIPT results earlier in pregnancy. Of the women receiving a normal NIPT result, 16 (2.4%) would rather have had invasive testing instead of NIPT; 11 because of the shorter waiting time and five because of more accurate results. One of the 18 women receiving a high-risk NIPT result for trisomies 21, 18 or 13 retrospectively would have preferred invasive testing as a follow up to FCT screening because of the shorter waiting time, and one woman would have preferred not to have had follow-up testing. None of the eight women receiving a high-risk NIPT result for other trisomies stated they would have preferred a different test.

### Future Offer of NIPT

Of all women, 54.3% thought that NIPT should be offered to all pregnant women irrespective of their risk for aneuploidy, while 36.7% thought that only those at high risk should be offered NIPT (either based on high-risk FCT (18%), previous pregnancy with aneuploidy (12.2%) or maternal age (6.5%)). 4.8% mentioned other target groups, for example those who have a close relative with aneuploidy.

## Discussion

This study shows that almost all high-risk pregnant women were glad to have been offered NIPT and, retrospectively, did not regret having NIPT, irrespective of NIPT result. The majority perceived the waiting time for NIPT results (mean 15 days) as (much) too long. After women had received a normal NIPT result they experienced significantly lower levels of general anxiety and child-related anxiety compared to the moment of intake for NIPT. Moreover, the majority of women felt reassured by the normal NIPT result and were confident it was correct.

Literature shows that having a high risk for aneuploidy significantly increases anxiousness in women (Lou et al. 2015), and this was also observed in the women participating in our study. After women received a normal NIPT result there was a significant reduction in anxiety to levels considered normal (Bekker et al., 2003; Green et al. 2004), although for 3% anxiety levels remained high. Residual anxiety in a small percentage of women has been shown in several studies on prenatal screening (Green et al. 2004). Most women in our study indicated they felt reassured by the normal NIPT result, and similar to the study of Vanstone et al. (2015), women felt confident the NIPT result was correct. Lewis et al. (2016) showed that reassurance was the main motivator for women to accept NIPT. Our study shows that a normal NIPT result is able to give most women this reassurance, and that the levels of increased anxiety generated by the high-risk FCT result are not sustained. The level of anxiety of women with a high-risk NIPT result for trisomy 21, 18 or 13 or for other trisomies remained high. The results also showed that some women with a normal NIPT result were not completely reassured about the results. At pre-test counseling, information on the limitations of NIPT was provided, including the residual risk of aneuploidy. It is therefore not unexpected that some women are not completely reassured after receiving a normal NIPT result. Moreover, one third of the women mentioned that NIPT did not give them sufficient certainty whether their child has a disorder, possibly indicating that they realize that NIPT cannot exclude all disorders in their child. A study by Wittman et al. (2016) however, showed that a normal NIPT result gave many women a false decrease in worry levels for conditions not screened for by NIPT. It demonstrates the importance to counsel women about the scope and limitations of NIPT, so they know how to accurately interpret the result.

Inadequate health literacy and having NIPT based on a medical indication were associated with less decrease in anxiety after receiving a normal NIPT result. Although these women still showed scores considered to be ‘normal anxiety’, it can be hypothesized that women with inadequate health literacy had more residual anxiety because they did not fully comprehend the test-result that stated that their normal NIPT result meant that the fetus most likely does not have trisomy 21, 18 of 13. Women with a medical indication (e.g. prior pregnancy with fetal trisomy) might have more residual anxiety because of their previous experience with having an affected child. There was no difference in anxiety levels at follow-up among women who did or did not make an informed choice for testing.

Similar to our study, Lewis et al. (2016) showed that women in the UK at increased risk for aneuploidy had a very positive experience with NIPT. Both studies show that the long turn-around time is perceived as a limitation. A Canadian study among women who had NIPT (waiting time results: 6–21 days) showed that the long waiting time for NIPT results is considered as very stressful (Vanstone et al. 2015). This is particularly true for women at a later gestational age who have little time left for a confirmative test in case of a high-risk NIPT result and a potential termination of pregnancy. Our study indicated that a waiting time for NIPT results up to ten days is acceptable for most women. Reducing turn-around time to this period could result in an even more positive experience with NIPT among pregnant high-risk women. Turn-around time decreased later in the study due to better equipment (Oepkes et al. 2016).

Test-results were preferably received by telephone and not by SMS, while almost a quarter of women did receive their result by SMS. This suggests that, even when results are normal, women would like a personal conversation with a healthcare professional, for example to ask additional questions.

Most women in this study believed that NIPT should be offered to all pregnant women irrespective of their risk for aneuploidy, as was also shown in other studies among pregnant women (Mikamo et al. 2015; van Schendel et al. 2015) and health professionals (Tamminga et al. 2015).

## Study Limitations and Research Recommendations

The results should be interpreted with some caution since the response rate to the post-test questionnaire was 57%. Besides the inability to perform subgroup analyses for women with a high-risk NIPT result, another limitation of this study is that there is a possibility of bias since very anxious women might have refrained from filling out the questionnaire. Moreover, women with low level of education and/or inadequate literacy responded less to the second questionnaire. Most women in our sample had a higher level of education. Dutch women are shown to be more likely to have prenatal screening if they are older and have above-average income (Gitsels-van der Wal et al. 2014). This could be due to the fact that this group often delays childbearing and are therefore more likely to be at increased risk for aneuploidy. The strength of this study was the large sample size and the prospective study design, which made it possible to study psychological outcomes over time. More specific research, including qualitative studies, is needed on the psychological effects of receiving a high-risk NIPT result for chromosomal aberrations other than trisomy 21, 18 and 13.

## Conclusion and Practice Implications

Introducing NIPT as an alternative to invasive testing in a national healthcare-funded prenatal screening program led to an offer that satisfied high-risk pregnant women. Those who received a normal result felt largely reassured and their anxiety levels returned to normal. Reducing waiting time for test-results is likely to improve women’s experiences with NIPT. Women who have inadequate health literacy and/or a medical history (i.e. previous child with aneuploidy) might benefit from extra explanation, or a more tailored approach or communication aids to support verbal communication, at pre-test counseling and after receiving normal NIPT results.

## Electronic supplementary material

ESM 1 (DOCX 21 kb)
