# Supplementary material for: Women’s Experience with Non-Invasive Prenatal Testing and Emotional Well-being and Satisfaction after Test-Results
Source: J Genet Couns. 2017 Jun 30;26(6):1348–56. doi: 10.1007/s10897-017-0118-3 (PMC5672853; doi:10.1007/s10897-017-0118-3)
Supplement: Supplementary file 1 — (DOCX 21 kb) [file 10897_2017_118_MOESM1_ESM.docx]

**SUPPLEMENTARY TABLES Journal of Genetic Counseling**

**Women’s Experience with Non-Invasive Prenatal Testing and Emotional Well-being and Satisfaction after Test-Results**

Rachèl V. van Schendel^1^, G.C.M. Lieve Page-Christiaens^2^, Lean Beulen^3^, Caterina M. Bilardo^4^, Marjon A. de Boer^5^, Audrey B.C. Coumans^6^, Brigitte H.W. Faas^7^, Irene M. van Langen^8^, Klaske D. Lichtenbelt^9^, Merel C. van Maarle^10^, Merryn V.E. Macville^11^, Dick Oepkes^12^, Eva Pajkrt^13^, Lidewij Henneman^1^ , for the Dutch NIPT Consortium

^1^Department of Clinical Genetics, Section Community Genetics and Amsterdam Public Health Research Institute, VU University Medical Center, Amsterdam, the Netherlands

^2^Department Obstetrics and Gynaecology, University Medical Center Utrecht, the Netherlands

^3^Department of Obstetrics and Gynaecology, Radboud University Medical Center, Nijmegen, the Netherlands

^4^Fetal Medicine Unit, Department of Obstetrics and Gynaecology, University Medical Center Groningen, University of Groningen, the Netherlands

^5^Department of Obstetrics and Gynaecology, VU University Medical Center, Amsterdam, the Netherlands

^6^Department of Obstetrics and Gynaecology, Maastricht UMC+, Maastricht, the Netherlands.

^7^Department of Human Genetics, Radboud University Medical Center, Nijmegen, the Netherlands

^8^Department of Genetics, University Medical Center Groningen, University of Groningen, the Netherlands

^9^Department of Medical Genetics, University Medical Center Utrecht, the Netherlands

^10^Department of Clinical Genetics, Academic Medical Center, Amsterdam, the Netherlands

^11^Department of Clinical Genetics, Maastricht UMC+, Maastricht, the Netherlands

^12^Department of Obstetrics, Leiden University Medical Center, Leiden, the Netherlands

^13^Fetal Medicine Unit, Department of Obstetrics and Gynaecology, Academic Medical Centre, Amsterdam, the Netherlands

**Corresponding author: Email:** [**l.henneman@vumc.nl**](mailto:l.henneman@vumc.nl)

**Table S1.** Details of women with a high risk (abnormal) NIPT result for trisomy 21,18 or 13, or for other trisomies, *N*=26.

| **NIPT result** | **Had confirmative testing?** | **Confirmative test** | **Result follow-up test** | **TOP** |
| --- | --- | --- | --- | --- |
| trisomy 21 | Yes | Amniocentesis | T21 confirmed | Yes |
| trisomy 21 | Yes | Amniocentesis | T21 confirmed | Yes |
| trisomy 21 | Yes | Amniocentesis | T21 confirmed | Yes |
| trisomy 21 | Yes | Amniocentesis | Normal result | No |
| trisomy 21 | Yes | Amniocentesis | T21 confirmed | Intends to have TOP |
| trisomy 21 | Yes | Amniocentesis | T21 confirmed | Intends to have TOP |
| trisomy 21 | Yes | Amniocentesis | T21 confirmed | Intends to have TOP |
| trisomy 21 | Yes | Amniocentesis | T21 confirmed | Intends to have TOP |
| trisomy 21 | Yes | Chorionic villus sampling | T21 confirmed | Intends to have TOP |
| trisomy 21 | Scheduled | Amniocentesis |  | Intends to have TOP |
| trisomy 21 | No | - | - | Not decided yet |
| trisomy 21 | No | - | - | No |
| trisomy 21 | No | - | - | No |
| trisomy 21 | No | - | - | No |
| trisomy 18 | Yes | Amniocentesis | T18 confirmed | Intends to have TOP |
| trisomy 18 | Yes | Amniocentesis | T18 confirmed | Intends to have TOP |
| trisomy 18 | Yes | Amniocentesis | T18 confirmed | Intends to have TOP |
| trisomy 13 | Yes | Amniocentesis | T13 confirmed | Miscarriage |
| trisomy 22 | Not decided yet | - | - | No |
| trisomy 20 | Not decided yet | - | - | Not decided yet |
| trisomy 16 | Yes | Amniocentesis | Normal result | No |
| trisomy 16 | Not decided yet | - | - | No |
| trisomy 16 | No | - | - | No |
| trisomy 9 | No | - | - | No |
| trisomy 7 | Not decided yet | - | - | No |
| trisomy 7 | No |  | - | No |

*TOP*= termination of pregnancy

**Table S2. Way of receiving test-result versus preferred method of receiving NIPT test-result, *N*=682**

|  | Preferred to receive test-result by: | | | | | | | |
| --- | --- | --- | --- | --- | --- | --- | --- | --- |
| Received test-result by: | **Telephone**  n (%) | **SMS**  n (%) | **Mail**  n (%) | **E-mail**  n (%) | **Patient Portal**  n (%) | **Consultation**  n (%) | **Other^b^**  n (%) | **Total**  n (%) |
| **Telephone** | 280 (41.4) | 1 (0.1) | 10 (1.5) | 5 (0.7) | 0 (0) | 23 (3.4) | 32 (4.7) | 351 (51.8) |
| **SMS** | 62 (9.2) | 60 (8.9) | 4 (0.6) | 9 (1.3) | 1 (0.1) | 16 (2.4) | 6 (0.9) | 158 (23.3) |
| **Mail** | 13 (1.9) | 3 (0.4) | 43 (6.4) | 11 (1.6) | 0 (0) | 3 (0.4) | 6 (0.9) | 79 (11.7) |
| **Email** | 0 (0) | 0 (0) | 0 (0) | 0 (0) | 0 (0) | 0 (0) | 0 (0) | 0 (0) |
| **Patient Portal** | 0 (0) | 0 (0) | 0 (0) | 0 (0) | 0 (0) | 0 (0) | 0 (0) | 0 (0) |
| **Consultation** | 0 (0) | 0 (0) | 0 (0) | 0 (0) | 0 (0) | 3 (0.4) | 0 (0) | 3 (0.4) |
| **Other^a^** | 25 (3.7) | 0 (0) | 1 (0.1) | 7 (1.0) | 0 (0) | 4 (0.6) | 49 (7.2) | 86 (12.7) |
| **Total** | 380 (56.1) | 64 (9.5) | 58 (8.6) | 32 (4.7) | 1 (0.1) | 49 (7.2) | 93 (13.7) | 677 (100) |

Numbers may not add up to the total due to missing values.

^a^Telephone and mail and/or e-mail; mail and consultation; contacted medical center themselves.

^b^Telephone and mail and/or e-mail; telephone and consultation; mail and consultation.
